# STAT3在肿瘤侵袭转移中的作用

**DOI:** 10.3779/j.issn.1009-3419.2010.10.09

**Published:** 2010-10-20

**Authors:** 志浩 吴

**Affiliations:** 300052 天津，天津医科大学总医院，天津市肺癌研究 所，天津市肺癌转移与肿瘤微环境重点实验室 Tianjin Key Laboratory of Lung Cancer Metastasis and Tumor Microenvironment, Tianjin Lung Cancer Institute, Tianjin Medical University General Hospital, Tianjin 300052, China

肿瘤转移是指恶性肿瘤细胞脱离原发肿瘤，通过各种转移方式，到达继发组织或器官得以继续增殖生长，形成与原发肿瘤相同性质的继发肿瘤的全过程。肿瘤的转移对肿瘤的发病率和死亡率起着很重要的作用^[1]^。虽然近些年来在癌症治疗方面有长足的进步，但是肿瘤转移仍然是临床工作中的一个巨大的难题。由于我们并不了解肿瘤细胞是如何从原位癌中散播开的，所以对于转移瘤的治疗目前尚存在很大的困难^[2]^。通常在转移瘤被确诊前肿瘤细胞已经从原位扩散到区域淋巴结，同时也有可能转移至远处器官^[3]^。虽然肿瘤转移每步过程的具体分子生物学机制尚未阐明，但其大体机制已经被初步认识^[4]^。第一步是肿瘤细胞获得侵袭的表型从而可以从原位癌中逃逸。肿瘤细胞还需要突破间质组织的边界，而这一过程又需要肿瘤细胞间黏附性降低以及细胞外基质的水解。之后还需介导新生血管生成并穿透血管内皮进入血液循环，一旦肿瘤细胞进入血液循环中，它必须要适应各种新的环境，如物理损伤，免疫监督以及因缺乏基质而产生的凋亡。最后又要穿过血管内皮而到达远端器官，适应不同的微环境从而最终形成转移灶^[5]^。其具体分子生物学基础包括相关基因的调控，黏附因子的改变，血管生成，基质金属蛋白酶与组织抑制剂和机体免疫状态的改变。

信号转导和转录激活因子3（signal transducer and activator of transcription 3, STAT3）是转录因子家族中的一员^[6]^。STATs家族由7个成员组成^[7]^：STAT1-STAT4、STAT5a、STAT5b及STAT6L^[8]^。研究表明，STATs家族在调节细胞增殖、存活、凋亡、分化及转移等方面都有很重要的作用，如STAT1可以通过抑制血管生成来产生抗肿瘤生成以及肿瘤转移的作用；STAT4、STAT6在肿瘤免疫逃逸方面有重要作用；STAT3持续活化又与多种肿瘤有关，如：卵巢癌、乳腺癌、前列腺癌、肺癌等等。近些年，其在转移中发挥的作用也渐渐得到重视。

## 1 STAT3的结构及其活化

STAT3在1994年作为一种DNA的结合因子而被发现，它可以结合在经过白介素-6（interleukin-6, IL-6）处理的肝细胞上与IL-6反应有关的急性期基因启动子上^[9]^。STAT3同时也被确定是一种结合在表皮生长因子启动子上的转录因子^[10]^。编码STAT3的基因定位于染色体17q21上，其DNA全长4 815 bp，含24个外显子。STAT3蛋白由750个-795个氨基酸组成，分子量约为89 kDa-92 kDa。其结构可分为6个功能区：N端的氨基酸保守序列、螺旋区、DNA结合域、连接区、SH2结构域和C端的转录活化区^[6]^。在C端存在705酪氨酸位点和727丝氨酸位点，它们磷酸化后可以处于激活状态。而STAT3又可以被很多细胞因子、生长因子、甚至是癌基因所激活，其中包括表皮生长因子^[11]^、血小板源性生长因子^[12]^、IL -6^[9]^、Src^[13]^和Ras^[14]^；此外，很多致癌物都可以引起STAT3信号通路的激活^[15]^。在酪氨酸705位点的磷酸化主要是受到受体型及非受体型蛋白酪氨酸激酶的调控，如表皮生长因子受体^[16]^、Src、Janus激酶（Janus kinase, JAK）^[17]^、细胞外信号调节蛋白激酶（extracellular signal-regulated kinase, ERK）^[18]^。STAT3单体在胞浆中磷酸化后，磷酸化的STAT3单体二聚化形成二聚体，变成有活性的STAT3。活化的STAT3进入核内，与其他的核蛋白结合直接调节基因的表达，最终影响肿瘤细胞的多种生物学功能^[19]^。

## 2 STAT3下游信号与肿瘤生物学

STAT3活化后可以激活很多其下游的基因表达。这些基因的表达对多种肿瘤的发病机制都有影响^[20]^。STAT3对肿瘤生物学的作用决定于STAT3的靶基因，它们都会在不同程度上调节肿瘤细胞的存活、生长、血管生成、侵袭以及免疫监督的逃逸^[21]^。STAT3的活化对于肿瘤细胞免于凋亡和促进增殖有重要的作用，它是通过调节抗凋亡以及促增殖的基因表达蛋白来实现的。这些基因包括*Bcl-xL*、*Mcl-1*、*Bcl-2*、*Fas*、*cyclin D1*、*survivin*和*c-Myc*^[22, 23]^。研究^[24]^表明，STAT3还可以通过结合p53启动子来抑制其转录从而影响p53介导的肿瘤细胞凋亡。此外还证实STAT3的激活还对核因子-κB（nuclear factor-kappaB, NF-κB）有调控作用^[25]^。

## 3 STAT3调控转移的相关机制

### 3.1 STAT3与基质金属蛋白酶

细胞外基质及基底膜的降解和破坏是肿瘤转移多阶段过程中的重要步骤之一，细胞外基质和基底膜主要组织结构可分为胶原、层粘素和纤维结合素等，这些组织结构的破坏和降解需要相应的溶解酶参加。因此基质金属蛋白酶在肿瘤侵袭和转移过程中担任了重要角色。STAT3激活后可以调控黑色素瘤细胞基质金属蛋白酶-2（matrix metalloproteinases-2, MMP-2）的基因转录活性^[26, 27]^，其中MMP-2对基底膜有降解作用^[28]^，这对许多恶性肿瘤的转移与侵袭都有着重要的作用。实际上，STAT3可以直接结合至MMP-2的启动子上，从而上调MMP-2转录的活性和蛋白水平^[27, 29, 30]^。如果在-617至-610位点上诱变高亲和性的STAT3位点，可以显著的降低STAT3诱导的MMP-2启动子的活性。经对比显示，STAT3活性缺失可以显著抑制MMP-2的表达、肿瘤侵袭、脑部和肺部的转移^[26, 27]^。最近研究^[31]^表明，在U87 MG人胶质瘤中，IL -6可以促进其侵袭性，高水平的STAT3可以增加MMP-2的表达与分泌。在膀胱癌中，可以通过调节基质金属蛋白酶-1（matrix metalloproteinases-1, MMP-1）和基质金属蛋白酶-10（matrix metalloproteinases-10, MMP-10）来发挥作用^[32]^。在乳腺癌中，通过基质金属蛋白酶-9（matrix metalloproteinases-9, MMP-9）发挥调节作用^[33]^。还有研究^[34]^表明，在人肺腺癌A549细胞系中，IL-6可以通过JAK/STAT3信号通路来增加MMP-10在细胞中的表达。

### 3.2 STAT3与上皮间质化

上皮间质化（epithelial to mesenchymal transition, EMT）是上皮细胞获得间质细胞特性的一种基本生物学过程^[35]^。EMT在肿瘤转移初始阶段有着重要的作用。最近的研究^[36]^表明，在MCF-7乳腺腺癌细胞系中，IL-6的异常表达和STAT3的激活可以使Twist持续表达，而Twist又与EMT有很密切的关系，从而激活乳腺癌细胞潜在的转移能力。STAT3可以在转录水平上调控Twist的活性。Twist是EMT主要调控位点之一。STAT3可以结合至Twist启动子近端第二个STAT3结合位点上，来调控它的转录活性。在晚期肿瘤中，p-STAT3（tyr705）与Twist水平有关^[37]^。另一项研究^[38]^表明，在胰腺癌细胞中，TGF-β诱导的肿瘤侵袭与STAT3的转录活性有关，且STAT3的活性受到Smad4的调控。Smad4可以引起STAT3的异常激活，从而导致转化生长因子-β（transforming growth factorbeta, TGF-β）在胰腺癌中产生促进肿瘤的作用。EGFR通路也可以通过STAT3介导*Twist*基因表达来诱导EMT的发生，这也为我们针对表皮生长因子受体（epidermal growth factor receptor, EGFR）以及STAT3靶向治疗提供了新的思路^[39]^。

### 3.3 STAT3与肿瘤血管生成

血管生成不仅对肿瘤原位生长有作用，而且对转移灶的生长也是有很重要的作用^[40]^。活化的STAT3可以调控肿瘤细胞内多因子和肿瘤内皮细胞中一些与血管生成有关的信号通路。在小鼠黑色素瘤中，发现血管内皮生长因子（vascular endothelial growth factor, VEGF）是STAT3的直接靶点，这也第一次证实STAT3在血管生成中的作用^[41]^。在胰腺癌动物模型中，通过显性缺失的STAT3来封闭STAT3的激活可以抑制血管内皮生长因子的表达，血管生成以及胰腺癌的肝转移^[42]^。STAT3还可以间接通过缺氧诱导性因子1α（hypoxia inducible factor-1α, HIF-1α）调控VEGF的转录^[43]^。还有报道^[26]^称STAT3可以调控其他血管生成因子，例如碱性成纤维细胞生长因子（basic growth fibroblast factor, bFGF），它可以在血管生成中参与肿瘤细胞的迁移、增殖以及内皮细胞的分化。在人黑色素瘤中调节STAT3活性可以在体内和体外明显的影响bFGF、VEGF、MMP-2这些血管因子的表达，从而影响黑色素瘤细胞的血管生成以及脑转移^[26]^。此外，STAT3还可以通过bFGF以及VEGF等在其受体发生的募集反应得到激活，将信号传导到内皮细胞，可见STAT3对内皮细胞的增殖、迁移和微血管形成有很重要的作用^[44, 45]^。IL-6可以通过活化STAT3促进血管内皮细胞迁移，从而使促进肿瘤血管生成及侵袭^[46]^。总之，许多促进血管生成的因子被证实是STAT3的靶基因，包括那些编码HIF-1、bFGFR、HGF、MMP-2以及MMP-9的基因^[47]^。

### 3.4 STAT3与肿瘤免疫逃逸

肿瘤细胞的增殖、存活、肿瘤血管生成和转移被视为评价肿瘤的几个的重要标志^[48]^。但是有证据显示免疫系统可以对肿瘤形成外部抑制^[49]^。侵袭与转移的发生需要抑制免疫监视系统。因此，很多研究表明，在罹患肿瘤的患者中免疫功能已经受损，这种情况又与肿瘤微环境有关^[50]^。

研究^[51]^表明STAT3可以对肿瘤细胞与宿主免疫系统产生互相影响。T细胞的稳定对正常免疫反应是很重要的，STAT3便是其中一条激活T细胞的信号通路。在一项关于B16小鼠黑色素瘤的基因治疗中，STAT3对肿瘤免疫逃逸的作用第一次得以证实^[52]^。含有显性失活型的STAT3蛋白载体接种到B16黑色素瘤小鼠皮下，在接种了STAT3蛋白那组小鼠中，肿瘤生长被有效抑制。这些结果表明STAT3不仅可以作为肿瘤治疗的靶点，还可以影响到与肿瘤细胞毗邻的细胞，从而扩大治疗作用并杀死肿瘤周围组织^[52]^。

STAT3对炎性反应有负相调控作用，对于那些缺乏*STAT3*基因的小鼠，给予它们巨噬细胞以及中性白细胞可以增强炎性反应^[53]^。在STAT3缺失的免疫细胞谱小鼠中，STAT3的活性对肿瘤细胞免疫逃逸有重要作用^[54]^。

STAT3已被证实在与肿瘤相关的免疫细胞中是持续活化的^[55]^，这种活化的的信号共同存在于肿瘤细胞与免疫细胞中，因此便可以促进肿瘤免疫逃逸的发生。抑制免疫细胞中STAT3的活性，便可以使其产生多种抗肿瘤的免疫作用，从而抑制肿瘤生长以及转移^[54]^。同样的，在造血过程中的骨髓细胞除去STAT3会导致炎性因子的过量产生，过量的骨髓细胞产生以及克隆恩病样的病理改变^[56]^。肿瘤相关巨噬细胞免疫抑制作用的增加与过表达的STAT3信号通路有很大关系^[57]^。总之，这些研究都表明STAT3信号通路在肿瘤细胞以及免疫细胞中激活，这样可以促进肿瘤免疫逃逸，如果应用靶定STAT3的药物可以产生抗肿瘤的免疫反应。

## 4 结语

总之，STAT3是一种可以介导多种细胞外刺激信号及酪氨酸激酶通路活性的转录因子，从而调控基因表达来影响细胞的多种重要功能。STAT3在许多生理过程中都扮演着重要的角色，如：细胞增殖、存活、自我更新及血管生成等。它是通过调节其他蛋白的表达来发挥在不同水平上调节作用的。但当正常调节过程遭到破坏时，STAT3则可能成为肿瘤转移中一个重要介质。STAT3的活化可以在不同程度上破坏细胞外基质以及造成基底膜的降解和破坏，这些都是肿瘤细胞发生早期转移时重要步骤。此外，STAT3还可以促进上皮细胞间质化的发生，从而促进肿瘤细胞由原位解离。同时，肿瘤血管生成在转移灶的生长也是有很重要的作用的，很多研究都表明STAT3在不同程度上促进转移灶肿瘤血管生成。最后，由于STAT3信号通路的特异性激活，影响了正常机体的免疫监督作用，得以使肿瘤细胞免于免疫系统的外部监视。因此，STAT3在肿瘤转移过程中扮演着重要的作用，STAT3抑制物的研发将是我们未来研究工作中一个重要的目标。
